# Neurocognition in stimulant addiction: reply to Robbins (2021)

**DOI:** 10.1093/psyrad/kkab010

**Published:** 2021-06-29

**Authors:** Benjamin Becker

**Affiliations:** University of Electronic Science and Technology of China, School of Life Science and Technology, China

On behalf of all authors who contributed to our recent article describing the results from a prospective longitudinal study demonstrating amphetamine-type stimulant- (ATS-) induced reductions in putamen volume and associated cognitive decline in moderate users of these drugs (Kendrick *et al*., 2021), I thank Professor Trevor Robbins for the flanking commentary. The commentary in the current issue of *Psychoradiology* (Robbins, 2021) sets our findings into the context of recent developments in the field of addiction research and I would like to take the opportunity to further elaborate on progress as well as challenges in neuroimaging of addiction.

Over recent decades, animal models and human neuroimaging studies have contributed tremendously to our understanding of the neurobiological basis of addiction leading to a reconceptualization of drug addiction as a chronic relapsing disorder of the brain. Most findings in humans are based on neuroimaging studies that use traditional case control designs comparing a single group of individuals with an established drug addiction or chronic drug use with a group of nonusing controls. Recent large scale meta-analyses of these case control studies demonstrated robust neurofunctional and neurostructural alterations in striatal–prefrontal circuits during reward and cognitive processing across addictions to different substances (Klugah-Brown *et al*., 2020a; Klugah-Brown *et al*., 2021; Luijten *et al*., 2017; Pando-Naude *et al*., 2021). As pointed out in the commentary by Robbins, although it is tempting to interpret the observed group differences in brain activity or morphology as ‘brain-based’ markers for addiction, the retrospective cross-sectional design of these studies does not allow for interpretation of the brain alterations in the addicted populations as neurobiological adaptations that specifically characterize or even critically mediate the transition to addiction. While limitations of the traditional retrospective case control design for psychiatric imaging in general have been increasingly debated (see also Etkin, 2019), the addiction imaging field has developed and implemented sophisticated experimental designs to overcome some of these limitations and to further disentangle behavioral and neural changes that accompany and probably mediate the transition to addiction from other alterations in addicted populations, including alterations that precede the onset of addiction or even the initiation of drug use, or characteristics that promote relapse after recovery, as well as changes related to the chronic exposure to potentially neurotoxic substances.

The endophenotype approach described by Robbins represents an excellent example of a sophisticated design that facilitated the determination of cognitive and brain vulnerability markers for addiction by including addicted individuals as well as their first-degree relatives, and demonstrated that structural brain alterations in inferior frontal and dorsal striatal regions, as well as impulsivity, may precede the onset of drug addiction, and thus render individuals at an increased risk of developing addiction (Ersche *et al*., 2013; Ersche *et al*., 2012). The implementation of cross-sectional designs that encompass additional groups to control for brain-based alterations not directly related to the addictive process has been generally a promising venue, such that studies including recreational drug users in addition to heavy drug users could demonstrate that a higher exposure to ATS was associated with lower gray matter volume in prefrontal regions (Daumann *et al*., 2011), or that exaggerated dorsal striatal reactivity in response to drug cues characterized cannabis- and alcohol-addicted individuals but not heavy recreational users of these substances (Vollstädt-Klein *et al*., 2010; Zhou *et al*., 2019). Prospective longitudinal designs in those beginning to use ATS have further contributed to determine brain-based and cognitive alterations that are likely to develop as a consequence of moderate ATS exposure (e.g. Schilt *et al*., 2007; Wagner *et al*., 2013), while structural brain alterations in the amygdala, dorsal striatum, and prefrontal cortex, as well as blunted neural reactivity in these circuits, may render occasional ATS users vulnerable to ultimately developing addictive patterns of use (Becker *et al*., 2015; Blair *et al*., 2018).

From a clinical perspective, the question arises as to whether behavioral and brain-based characteristics not only render individuals at a greater risk of initiating substance use and developing an addiction, but also whether specific characteristics may allow prediction of relapse following successful treatment and recovery. In line with the proposed role of the striato-frontal circuits in several stages of the addictive process, prospective designs in recently abstinent ATS users suggest that insular and striatal responses can differentiate individuals who subsequently relapse from those who will not (e.g. Stewart *et al*., 2014). From a basic science perspective, the question arises, to what extent do the observed brain changes in drug addicted individuals ultimately reflect the addictive process per se? Increasing research on behavioral addictions, particularly pathological gambling or internet gaming disorder, may contribute to addressing this question given that these populations may allow examination of the development of addictive and compulsive behavior in the absence of primary substance-induced neuroadaptations or potential neurotoxic effects of chronic substance exposure. However, while general conceptualizations and initial prospective studies suggest an involvement of the fronto-striatal circuitry in excessive internet gaming (Brand *et al*., 2019; Zhou X *et al*. 2019; Zhou X *et al*., 2020), other prospective studies and large-scale meta-analyses failed to find an engagement of these circuits (Klugah-Brown *et al*., 2020b; Yu *et al*., 2021), suggesting that despite similar symptoms on the behavioral level, partly distinct neural mechanisms may underpin substance and behavioral addictions (see also Clark *et al*., 2019 with respect to pathological gambling).

Progress in neuroimaging of addiction thus uncovered a comprehensive and complex involvement of the fronto-striatal circuits in addiction, ranging from vulnerability markers toward changes that may critically mediate the transition to compulsive drug taking, which are likely to interact on different levels, such that, for example, a predisposing stronger reliance on dorsal striatum-dependent habitual learning or impaired prefrontal regulatory control may prospectively increase the risk of addiction, and become progressively further dysregulated with chronic substance exposure and addiction-related neuroplastic maladpatations (see also commentary by Robbins, 2021).

Progress with respect to the accurate determination of the neurobiological markers of addiction in humans will, however, also depend on the robustness and specificity of the imaging-based markers, with variability in the identified neurobiological markers due to variability in the different processing pipelines (e.g. Zhou X *et al*., 2021), as well as a low spatial specificity and effect sizes of the conventional neuroimaging analytic approaches representing particular challenges (e.g. Poldrack *et al*., 2017). The pace of progress in neuroimaging of addiction will therefore also depend on the implementation of reproducible and robust neuroimaging practices and the implementation of more complex brain modelling approaches (Woo *et al*., 2017), which may allow a more complete description of process-specific neurofunctional representations (e.g. Zhou F *et al*., 2020) and may facilitate progress in translational biomarker development (Woo *et al*., 2017).
